# Investigating the Electronic Properties and Stability of Rh_3_ Clusters on Rutile TiO_2_ for Potential Photocatalytic Applications

**DOI:** 10.3390/nano14121051

**Published:** 2024-06-19

**Authors:** Moteb Alotaibi

**Affiliations:** Department of Physics, College of Science and Humanities in Al-Kharj, Prince Sattam Bin Abdulaziz University, Al-Kharj 11942, Saudi Arabia; mot.alotaibi@psau.edu.sa

**Keywords:** Rh_3_ clusters, TiO_2_ (110), green hydrogen generation, DFT, oxygen vacancy

## Abstract

Addressing the pressing needs for alternatives to fossil fuel-based energy sources, this research explores the intricate interplay between Rhodium (Rh_3_) clusters and titanium dioxide (TiO_2_) to improve photocatalytic water splitting for the generation of eco-friendly hydrogen. This research applies the density functional theory (DFT) coupled with the Hartree–Fock theory to meticulously examine the structural and electronic structures of Rh_3_ clusters on TiO_2_ (110) interfaces. Considering the photocatalytic capabilities of TiO_2_ and its inherent limitations in harnessing visible light, the potential for metals such as Rh_3_ clusters to act as co-catalysts is assessed. The results show that triangular Rh_3_ clusters demonstrate remarkable stability and efficacy in charge transfer when integrated into rutile TiO_2_ (110), undergoing oxidation in optimal adsorption conditions and altering the electronic structures of TiO_2_. The subsequent analysis of TiO_2_ surfaces exhibiting defects indicates that Rh_3_ clusters elevate the energy necessary for the formation of an oxygen vacancy, thereby enhancing the stability of the metal oxide. Additionally, the combination of Rh_3_-cluster adsorption and oxygen-vacancy formation generates polaronic and localized states, crucial for enhancing the photocatalytic activity of metal oxide in the visible light range. Through the DFT analysis, this study elucidates the importance of Rh_3_ clusters as co-catalysts in TiO_2_-based photocatalytic frameworks, paving the way for empirical testing and the fabrication of effective photocatalysts for hydrogen production. The elucidated impact on oxygen vacancy formation and electronic structures highlights the complex interplay between Rh_3_ clusters and TiO_2_ surfaces, providing insightful guidance for subsequent studies aimed at achieving clean and sustainable energy solutions.

## 1. Introduction

Hydrogen has garnered remarkable attention as a sustainable energy source, owing to its high energy yield and potential for reducing environmental pollution. As a fuel, hydrogen is particularly appealing because its combustion results in water (H_2_O), eliminating the emissions of carbon dioxide (CO_2_) and other harmful pollutants, which are commonly associated with the burning of fossil fuels [1]. This characteristic renders hydrogen a fundamental element in strategies designed to diminish greenhouse gas emissions and alleviate the effects of climate change. The energy content of hydrogen is another notable aspect. It possesses nearly three times the energy per unit weight compared to gasoline, making it an extremely efficient fuel source [2]. This high energy density is especially advantageous for applications that demand significant energy inputs, such as transportation and industrial processes. Diverse methods are available for producing hydrogen, including from biomass, nuclear power, natural gas, and renewable power sources such as solar and wind energy [3]. This flexibility in production enhances energy security and facilitates the integration of hydrogen into various energy systems. In the automotive industry, hydrogen’s utilization in fuel cells is a key area of interest. Fuel cells efficiently convert hydrogen’s chemical energy into electricity, offering a cleaner and more efficient alternative to traditional internal combustion engines [4]. Despite its vast potential as an energy carrier, hydrogen’s widespread adoption faces several challenges. These challenges include the need for extensive infrastructure development, high production costs, and the need for further technological advancements in efficient hydrogen production, storage, and transportation systems [5].

Given the extensive potential of hydrogen as a sustainable and efficient energy source, it is crucial to explore innovative methods for its production. Photocatalysis represents one such method, offering a sustainable approach to hydrogen generation by utilizing solar energy to split H_2_O molecules. This method enhances hydrogen’s contribution to lowering greenhouse gas emissions and lessening the effects of climate change, while also tackling the hurdles in hydrogen production, including the demand for renewable and economical production methods. Photocatalysis is a process that leverages light to accelerate a chemical reaction, a phenomenon that has gained substantial attention in the field of renewable energy, particularly for hydrogen production. At its core, photocatalysis involves the absorption of light by a photocatalyst, which then generates electron–hole pairs that can drive chemical reactions. This procedure is crucial for utilizing solar power to separate water into hydrogen and oxygen through photocatalytic water splitting. It represents a hopeful avenue for the sustainable production of hydrogen, in line with worldwide initiatives to foster clean and renewable energy solutions.

The process of photocatalysis starts when a semiconductor, often a photocatalyst such as titanium dioxide (TiO_2_), captures photons with energy that matches or exceeds its band gap. This process involves the promotion of electrons from the valence band (VB) to the conduction band (CB), resulting in the creation of electron–hole pairs. These photogenerated charge carriers can then participate in redox reactions on the surface of the catalyst. In the context of hydrogen production, the excited electrons reduce protons in water to hydrogen, while the holes oxidize water or hydroxide ions to oxygen [6,7]. The effectiveness of photocatalytic hydrogen production is heavily influenced by the characteristics of the photocatalyst, such as its capacity to absorb light, create electron–hole pairs, and allow their efficient separation and transmission to the reactants. TiO_2_, for instance, is widely used owing to its stability [8], non-toxicity [9], and strong oxidative power. However, its band gap only allows for the absorption of ultraviolet light, which limits its efficiency under solar irradiation [10]. To overcome this limitation, extensive research has focused on modifying TiO_2_ with various strategies, such as introducing metal or non-metal elements [11,12], combining it with other semiconductors [13,14], and enhancing it with organic dyes [15], to broaden the light absorption of the material into the visible spectrum [16]. The (110) surface of rutile TiO_2_ is the most thermodynamically stable and energetically favorable crystallographic face of rutile TiO_2_ [17,18]. This surface is characterized by a distinctive arrangement of titanium (Ti) and oxygen (O) atoms, forming a pattern of alternating rows of exposed Ti and bridging O atoms along the (110) direction. The Ti atoms are six-coordinate, bonded to six oxygen atoms, whereas the bridging oxygen atoms are three-coordinate, bonded to three titanium atoms. This asymmetrical coordination creates distinct active sites for chemical reactions on the surface [19]. The surface properties of rutile TiO_2_ (110) are also influenced by the presence of defects, such as oxygen vacancies. These vacancies can serve as places where charge carriers can become trapped, which in turn affects the process of recombination of electron–hole pairs.

Numerous studies have focused on depositing various metals onto TiO_2_ to enhance its photocatalytic efficiency. The adsorption of metal particles on TiO_2_ surfaces has been a subject of extensive research, primarily due to the potential enhancements in the photocatalytic activities that these metals can provide. Various metals, including noble metals such as platinum (Pt) [20,21], gold (Au) [22], ruthenium (Ru) [23], silver (Ag) [24,25], a precious metal such as rhodium (Rh) [26,27,28,29], as well as transition metals such as copper (Cu) [30,31,32] and iron (Fe) [33], have been studied for their effects on TiO_2_ photocatalytic performance. For instance, Rh clusters, specifically Rh_3_, have been thoroughly investigated because of their distinctive electrical and geometric characteristics, which greatly enhance catalytic activity. Majumdar and Balasubramanian [34] investigated the interaction between CO and Rh_3_ clusters, offering valuable insights into the electronic states and bonding properties of these small clusters. The researchers showed that Rh_3_ clusters have several stable geometries, with different electronic states. These variations are important for catalytic processes because the clusters can enable various types of chemical interactions based on their adsorption configuration.

Additionally, we selected Rh_3_ clusters for our investigation based on their proven stability and efficiency in facilitating charge transfer processes, as emphasized by Majumdar and Balasubramanian. These attributes are crucial for augmenting the photocatalytic efficacy of TiO_2_ in the production of hydrogen. The capacity of Rh_3_ to alter the electrical configuration of TiO_2_, thereby enhancing its light absorption and diminishing the recombination of charge carriers, is in line with our objective to create more effective photocatalysts. In addition, Rh_3_ clusters are recognized for their capacity to function as co-catalysts by offering active sites that enhance the hydrogen evolution reaction (HER). This claim is substantiated by literature demonstrating that the presence of small Rh clusters can greatly augment the photocatalytic efficiency of TiO_2_ when exposed to visible light, as evidenced by a study conducted by Wang et al. [28]. Considering these factors, Rh_3_ clusters were chosen based on their established catalytic capabilities and their particular affinity for TiO_2_, which has the potential to enhance photocatalytic performance. Utilizing Rh_3_ exploits the distinctive characteristics of TiO_2_-based systems and supports novel strategies to improve the efficiency of converting solar energy to hydrogen.

Further, Rh and niobium (Nb) codoped TiO_2_ nanorods demonstrate significant visible light absorption and effective separation of photogenerated carriers. As a result, this photocatalyst exhibits extremely high efficiency in generating hydrogen when exposed to either UV or visible light [35]. Camposeco et al. [36] found that the RhCu/TiO_2_ oxide structure had a much superior photocatalytic hydrogen evolution performance, approximately twice as high as that of the Cu/TiO_2_ monometallic photocatalyst. The exceptional performance of this system can be attributed to the efficient transfer of charge carriers and the delayed recombination of electrons and holes, which is a result of the inclusion of Rh. Similarly, Subramanian et al. [37] demonstrated enhanced photoinduced charge separation with Au-deposited TiO_2_. Furthermore, various metals have also been explored for their ability to alter the electronic structures of TiO_2_. Incorporating these metals can establish additional energy states within the band gap of TiO_2_ or modify its surface characteristics, thereby improving its capacity to absorb visible light and engage in photocatalytic processes. For example, it has been reported that the superior photocatalytic activity in TiO_2_ incorporated with Cu was attributed to the reduction of the band gap and the introduction of impurity levels that facilitate visible light absorption [38].

Upon being applied to a TiO_2_ surface, Rh nanoparticles can serve as electron traps, capturing the electrons produced by light from the semiconductor. This action aids in the effective separation of electron–hole pairs, an essential aspect in enhancing the effectiveness of photocatalytic activities. The separation of charge carriers minimizes the recombination losses and ensures that more electrons are available for the hydrogen evolution reaction (HER) in the H_2_O splitting processes. Moreover, the presence of Rh on TiO_2_ has been shown to modify the photocatalyst’s light absorption characteristics. Rh can create new energy levels in the band gap of TiO_2_, enabling the photocatalyst to absorb a broader spectrum of light, including visible light. This modification is significant because it allows for the use of a larger segment of the solar spectrum, thereby enhancing the photocatalyst’s overall solar-to-hydrogen efficiency. It has also been shown that SrTiO_3_ photocatalysts coated with Rh demonstrate boosted photocatalytic efficiency under irradiation by both visible and UV light, in comparison to the undoped material [39]. 

Another critical aspect of Rh deposition is forming energetic sites for HER. The Rh nanoparticles can serve as catalytic sites where hydrogen ions (protons) are efficiently reduced to hydrogen gas. This localized catalytic activity can significantly accelerate the rate of hydrogen production. Rh is considered a noble metal that has become an important catalyst in the field of photocatalytic hydrogen evolution reaction (HER). This is because it has a high work function and favorable Gibbs adsorption energies for hydrogen atoms [40]. M. Alotaibi [41] conducted a DFT calculation to investigate the impact of Rh_5_ nanoclusters on the photocatalytic efficiency of a perfect and reduced rutile TiO_2_ for green hydrogen generation. It was reported that the Rh_5_ nanoclusters oxidized, donating their unpaired charge to the substrate, leading to enhanced activity. It was also found the Rh_5_ clusters stabilize the catalyst and increase the oxygen formation energy. The existing literature has examined the role of metal clusters in enhancing photocatalytic materials, yet the distinct interactions and impacts of Rh_3_ clusters on perfect and defective rutile TiO_2_ (110) surfaces remain insufficiently investigated. As a result, this study, implementing complex DFT methods, seeks to enrich the current understanding of these specific interactions. This research focuses on elucidating the implications of these interactions for the improvement of photocatalytic performance, particularly under conditions of solar irradiation.

This research delves into the study of Rh_3_ clusters on both stoichiometric and defective rutile TiO_2_ (110) surfaces, using DFT for its examination. The DFT-D3 methodology is chosen due to its demonstrated efficacy in accurately representing the adsorption phenomena of Rh_3_ clusters on rutile TiO_2_ surfaces. Additionally, this study incorporates the HSE06 hybrid function, formulated by Heyd, Scuseria, and Ernzerhof [42], to scrutinize the electronic characteristic relevant to the formation of polaron on TiO_2_ surfaces. This function is particularly distinguished for incorporating a fraction of exact exchange, thereby surpassing the capabilities of conventional DFT methods in depicting electronic properties. The structure of the article is methodically organized: Section 2 provides an exhaustive description of the simulation methodologies, aiming to ensure clarity and replicability in the research method. Section 3 presents and critically analyzes the results obtained from the simulations. This section delves into the interactions and dynamics within the system under study and investigates the concept of polaron, comparing these new insights with previous research to augment the conceptual comprehension of charge transfer on TiO_2_ surfaces. The concluding Section 4 encapsulates the primary results of this study, articulating their broader implications for the scientific domain, particularly in relation to the field of renewable energy. The objective of this study is to improve the comprehension of the photocatalytic characteristics of Rh_3_ clusters attached to TiO_2_ surfaces, with a specific emphasis on the rutile TiO_2_ (110) surface. It also aims to assess the catalytic efficiency of Rh_3_ clusters as potential co-catalysts for the process of photocatalytic water splitting, which is crucial for the sustainable production of hydrogen.

## 2. Computational Details

The analysis and understanding of the electrical properties and photon absorption capabilities of Rh_3_ clusters present a significant challenge in the field of computational materials research. This investigation addresses this complexity through the application of complicated computational simulations, adopting a comprehensive methodology to accurately depict and examine these critical attributes. Through the combination of DFT and the HSE06 hybrid function, the electronic configuration of Rh_3_ clusters is meticulously explored. The purpose of this modelling is to acquire a comprehensive understanding of the interactions occurring within the clusters, thereby elucidating the role of each atom in contributing to the electronic properties of the cluster. Furthermore, the potential utility of Rh_3_ clusters in the field of photocatalysis is scrutinized, with a particular emphasis on their unique electronic characteristics and their capacity to enhance the efficiency of light absorption in rutile TiO_2_.

This simulation methodology investigation is anchored in the deployment of the Vienna Ab initio Simulation Package (VASP) [43,44,45], which employs the HSE06 hybrid exchange-correlation function, noted for its precision. This function adeptly integrates the short- and long-range components of the Perdew–Burke–Ernzerhof (PBE) [46] exchange function and a short-range component of the Hartree–Fock (HF) theory, facilitating a detailed and accurate exploration of correlation effects and electron exchange. Furthermore, this study leverages the projector augmented wave (PAW) technique [47,48] and PAW-PBE pseudopotentials to meticulously model the interplays between valence electrons and ion cores, which is crucial for determining the clusters’ electronic characteristics. The valence electrons include the electron shells of O (2s, 2p), Rh (4d, 5s) and Ti (3s, 4s, 3p, 3d), providing a nuanced understanding of the electronic environment. To overcome the typical drawbacks of conventional DFT approaches, notably the self-interaction error leading to spurious electron delocalization, this research adopts a generalized gradient approximation (GGA), supplemented with a Hubbard parameter (U). The chosen U value for the third orbitals of Ti is 4.2 eV, aligning with findings from prior research [30,49,50], thereby enhancing the accuracy of the simulations concerning the electronic attributes of the clusters.

This study encompasses an extensive simulation of the stoichiometric rutile TiO_2_ (110), which is essential to understanding the interplays between the Rh_3_ clusters and this particular surface. To precisely model isolated Rh_3_ clusters, it is essential to employ large supercells i.e., 30 Å × 30 Å × 30 Å. The rutile surface is represented using a unit cell with 12 Å × 13 Å, which includes a vacuum layer of 20 Å. This approach is aimed at accurately reflecting the surface structure frequently encountered in experimental studies. This measure is critical for eliminating any inadvertent interactions with periodic images, thereby ensuring the precision of energy calculations. The selection of the simulation parameters is executed with meticulous care to achieve an equilibrium between computational efficiency and precision. In this work, a single k-point is used, which is chosen due to the huge dimensions of the supercell used. By reducing the size, the Brillouin zone is practically diminished to a point, making it possible to adequately depict the electronic structure of large systems using a single k-point, usually known as the Gamma point. This methodology is especially beneficial in investigations that focus on surfaces or huge supercells, as it helps to decrease the impact of boundary effects. The implementation includes a Gaussian smearing of 0.05 eV for the occupancy of bands and the determination of the cut-off energy for the plane waves basis set at 500 eV. The adherence to a self-consistent electronic minimization technique, with a relaxation force criterion of 0.02 eV/Å and a convergence criterion of 10^−4^ eV, guarantees the accuracy and stability of the modeled structures.

The application of van der Waals (vdW) corrections [51] via the spin-polarized PBE scheme, augmented by the Becke–Jonson damping function [52], constitutes a critical component in the nuanced examination of the metal–oxide interactions. This incorporation transcends basic computational methodology, proving essential for the precise delineation of the intricate physicochemical interactions that significantly impact the reactivity and stability of nanomaterials. The calculation of the adsorption energy ($E_{ads}$) for Rh_3_ clusters, requiring detailed energy analysis, serves a dual purpose. It not only facilitates an evaluation of the adsorption stability but also illuminates the clusters’ potential in catalytic applications. The quantitative assessment of the Rh_3_ clusters’ adsorption stability is conducted through the calculation of their adsorption energy, employing the subsequent equation:

(1) $$E_{ads}=E_{tot}-E_{TiO_{2}}-E_{Rh_{3}}$$

In this context, $E_{tot}$ signifies the total energy of the integrated material, whereas $E_{TiO_{2}}$ and $E_{Rh_{3}}$ are the final energies of the TiO_2_ and Rh_3_ clusters, respectively. Moreover, the energy pertinent to the genesis of oxygen vacancies ($E_{Vo}$) is determined through the application of the subsequent equation:

(2) $$E_{Vo}=E_{surface+Vo}+\frac{1}{2}E_{O_{2}}-E_{surface}$$

Within this equation, $E_{surface+Vo}$ denotes the total energy of the TiO_2_ with an oxygen vacancy, $E_{O_{2}}$ is the total energy of the oxygen dimer in the gas phase, and $E_{surface}$ is the total energy of the perfect TiO_2_. The construction and visual depiction of the configurations presented in this research were executed via the application of VESTA [53].

## 3. Results and Discussion

### 3.1. Rh_3_ Cluster

**Figure 1:**
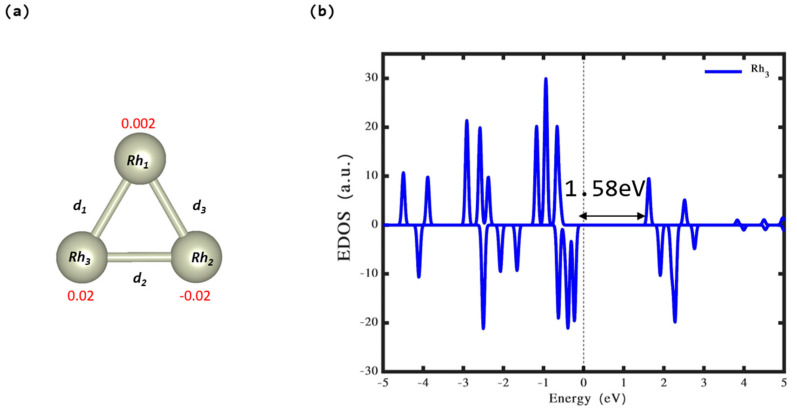
(**a**) The stable configuration of the Rh_3_ cluster and (**b**) density of states (DOS). Within the illustration, the electron quantity on each atom is denoted by red figures. The symbols d_1_, d_2_, and d_3_ are utilized to represent the distances of the Rh–Rh bonds in the cluster. The vertical line set at zero represents the Fermi energy.

The findings depicted in Figure 1a,b offer profound understanding of the structural and electrical characteristics of the Rh_3_ cluster in its gas phase. The stable structure of the cluster, which adopts a triangular shape in a doublet state, exhibits distinctive stability features and electronic responses. It has been established that the triangular formation of the Rh_3_ cluster represents its most stable configuration. The gas-phase stability of the Rh_3_ cluster is notably highlighted by its total energy measurement of −11.56 eV. A detailed geometrical examination of the Rh_3_ cluster reveals an equality in the bond lengths (d_1_, d_2_, and d_3_), measured at 2.36 Å. The bond lengths, being uniformly measured at 2.36 Å, are particularly interesting. This uniformity leads to a stable structure. In metal clusters, the bond lengths can significantly affect the physical and chemical properties. Uniform bond lengths in a triangular cluster such as Rh_3_ suggest that each Rh–Rh interaction contributes equally to the cluster’s stability, which might not be the case in clusters with varying bond lengths. Given its stability and unique electronic characteristics, the Rh_3_ cluster could have potential applications in catalysis and electronic materials. For example, its unpaired electrons might make it a good candidate for facilitating reactions that require single-electron transfers.

The analysis of the density of states offers additional clarification on the electronic properties of the cluster. The band gap is a fundamental electronic property that describes the energy difference between the VB and the CB in semiconductor materials. A band gap of 1.58 eV for the Rh_3_ cluster suggests that it behaves as a semiconductor. This is an important result because it indicates the potential utility of the cluster in electronic or photocatalytic applications, such as photovoltaic cells or water splitting. The size of the band gap also gives insights into the electronic conductivity and optical properties of the material: a band gap in this range suggests that the cluster might absorb and emit light within the spectrum ranging from visible to near-infrared wavelengths. Moreover, the Bader charge information yields crucial understanding regarding the electron distribution across the cluster. This analysis is pivotal for understanding how electrons are localized around the atoms, which in turn affects the chemical reactivity and interactions of the clusters with other molecules. Specifically, the Rh_2_ and Rh_3_ atoms in the cluster are characterized by electronic charges of −0.02 e^−^ and 0.02 e^−^, correspondingly. The charge on Rh_2_ atom indicates a slight excess of electrons on the Rh_2_ atom, making it slightly negatively charged. This negative charge could affect the chemical reactivity of the cluster, potentially making the Rh_2_ atom a site for attracting positively charged species (cations) or acting as an active site in catalysis. Conversely, Rh_3_ being positively charged by an equivalent amount suggests a slight deficiency of electrons. This positive charge might make the Rh_3_ atom more susceptible to attracting negatively charged species (anions), altering the reaction of the cluster with other molecules, as well as its photocatalytic properties. The minimal positive charge on the Rh_1_ atom suggests a near-neutral electronic environment. This subtle difference in charge, compared to the Rh_2_ and Rh_3_ atoms, could lead to a differentiated behavior in chemical reactions or interactions, although the effect might be less pronounced due to the small magnitude of the charge.

### 3.2. Rh_3_ Adsorbed on Stoichiometric TiO_2_

**Figure 2:**
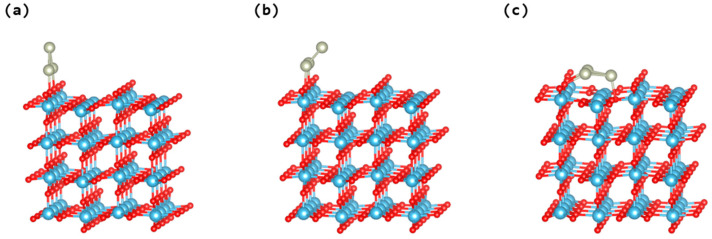
Different adsorption shapes of triangular Rh_3_ clusters on the rutile TiO_2_ (110) surface, including (**a**) the upright cluster, (**b**) the tilted Rh_3_ (aligned parallel to the substrate), and (**c**) the tilted Rh_3_ (aligned parallel to the substrate). The Ti, Rh, and O atoms are represented by blue, grey, and red balls, respectively.

The projected density of states for the stoichiometric rutile TiO_2_ (110) surface was calculated, revealing a band gap of approximately 3.16 eV, as shown in Figure S1. This estimation roughly corresponds to prior empirical findings [10]. Advancing the research to encompass the structural and electronic features of Rh_3_ clusters adsorbed on pristine and reduced TiO_2_ rutile (110) surfaces constitutes a significant progression towards elucidating the interactions between the oxide surface and the clusters. The comprehensive computational analysis of three unique adsorption schemes for triangular Rh_3_ clusters demonstrates the impact of orientation on the stability of the clusters and their interactions with the surface of TiO_2_ (see Figure 2). The consistent observation that Rh_3_ clusters maintain their structure without distortion upon adsorption underscores a robust and enduring synergy with the surface of TiO_2_. Of particular interest is the result that the vertical orientation of the Rh_3_ cluster (in Figure 2a) is more stable than the configuration that is tilted (parallel to the substrate, as depicted in Figure 2b), with a stability discrepancy of approximately 0.20 eV. This result diverges from our previous research involving Ag_5_ and Rh_5_ clusters [25,41], which indicated a greater stability in the tilted structures.

Moreover, the adsorption configuration shown in Figure 2c, where the Rh_3_ cluster is bound to three oxygen atoms on the TiO_2_ surface, has significantly greater stability, with an adsorption energy of −4.36 eV compared to both the upright and the tilted cluster orientations (see Figure 2a,b). This arrangement leads to a more intense contact and stronger bonding, which greatly boosts the stability and electrical characteristics of the system, hence boosting the overall photocatalytic performance. This observation of a superior stability, underscored by an average Rh–O bond distance of about 2.08 Å, shows a more effective interaction between the cluster and the substrate. This insight is pivotal in understanding the dynamics that govern the adsorption and stability of metal clusters on TiO_2_ surfaces, which has profound implications for catalysis and materials engineering. An in-depth comparative analysis provided in Table 1 elaborates on the adsorption energy and the related charges for the various adsorptions of Rh_3_ clusters, offering a comprehensive overview of the impacts of orientation on the stability of clusters and their interaction with the TiO_2_ surface.

**Figure 3:**
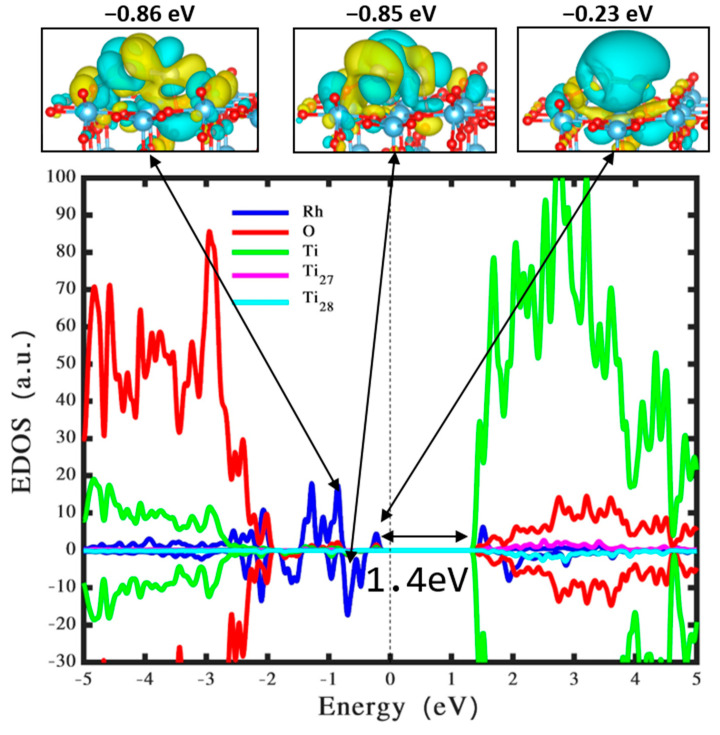
The projected density of states and the wavefunction for the optimum Rh_3_ cluster on a stoichiometric TiO_2_ (110) surface. The curves related to Ti, Rh, and O atoms are denoted by green, blue, and red, correspondingly.

The charge transfer, quantified at nearly +0.56 e^−^, from the Rh_3_ to the TiO_2_ surface in its most stable form, suggests the Rh_3_ cluster is oxidized. This movement of electrons is consistent with findings from previous studies [27,54] and occupies a pivotal position in comprehending the dynamics of interaction between the cluster and the surface. The application of the HSE06 function for the projected density of states analysis, as well as wavefunction calculations for the configuration depicted in Figure 2c (shown in Figure 3), yielded noteworthy insights. Specifically, the incorporation of the Rh_3_ cluster onto the TiO_2_ (110) surface induces the formation of intragaps within the band gap, an important element in modifying the electronic attributes of the TiO_2_ surface, which may influence its photocatalytic efficiency.

The localization of the highest occupied molecular orbital (HOMO) of the Rh_3_ cluster at an elevated energy level (−0.23 eV, roughly 1.4 eV beneath CB edge) highlights the substantial impact of the Rh_3_ cluster on the electronic structures of the TiO_2_ surface. The location of the HOMO suggests that the Rh_3_ cluster brings energy levels within the energy gap of TiO_2_. This can lead to increased absorption of visible light and improved separation of the charge carriers created by light, which is essential for photocatalytic activity. In our prior investigation of Rh_5_ clusters on TiO_2_ [41], we observed that the HOMO of the Rh_5_ cluster was located around 1.2 eV below the CB edge. This positioning is closer to the CB compared to Rh_3_ clusters. The closer proximity of the Rh_5_ clusters implies that they may enhance charge transfer and accelerate the electron injection into the CB of TiO_2_. This could result in distinct photocatalytic activities. The comparison reveals that both Rh_3_ and Rh_5_ clusters alter the electronic structure of TiO_2_. However, their distinct HOMO positions lead to differing effects on photocatalytic performance. Rh_3_ has the capacity to absorb a wider range of light, while Rh_5_ facilitates more direct pathways for charge transfer. Additionally, the emergence of a state at −0.85 eV, associated with the electron donation from the Rh_3_ cluster to the metal oxide’s surface, is clearly noted by the wavefunction. The establishment of mid-gap states resulting from the electron migration from the Rh_3_ cluster is instrumental in augmenting photon absorption efficiency across both the ultraviolet and visible spectra [31,55]. This aspect is crucial for photocatalytic processes, expanding the spectrum of light that could be harnessed in photocatalytic activities. Furthermore, the anchoring of the Rh_3_ onto the TiO_2_ surface leads to the rearrangement of the CB, bestowing metallic characteristics upon the substance. Similar alterations in electronic structures have been documented in TiO_2_ combined with Ag_3_ and Ag_5_ clusters [24]. The arrangement of intragaps to capture electrons from the VB under visible light exposure, due to the diminished energy gap, promotes electron transport, which is vital for improved photocatalytic hydrogen generation [56]. Recently, Wang et al. [28] conducted an experiment and found that the efficiency of hydrogen evolution through photocatalysis was approximately fifty times higher when using Rh-doped rutile compared to Rh-doped anatase powders.

### 3.3. Rh_3_ Adsorbed on Reduced TiO_2_

This study advances our understanding of the dynamic interactions between Rh_3_ clusters and TiO_2_, highlighting the significant role of Rh_3_ clusters in fostering the formation of oxygen vacancies, especially within defective TiO_2_ frameworks. In our preceding investigation [25], the findings substantiated that the energy needed to induce an oxygen vacancy on perfect TiO_2_ surfaces are approximately 0.59 eV lower, compared to generating a sub-surface vacancy. This result is in agreement with earlier research [57,58] and is confirmed by the evidence presented in Table S1 and Figure S2. A pivotal element of this study entailed the exploration of the highest stability the Rh_3_ on the TiO_2_ surface, as depicted in Figure 2c, and its influence on photocatalytic activity, mainly concerning the generation of surface oxygen vacancies. The results demonstrate that the integration of the Rh_3_ cluster onto the TiO_2_ surface enhances stability, as indicated by a 0.10 eV rise in the energy needed to create an oxygen vacancy. This elevation points to a more robust surface stability in the presence of the Rh_3_ cluster. Table S1 and Figure S3 offer comparative insights into this trend. In addition, our investigation revealed a reduction in the adsorption energy of Rh_3_ clusters on TiO_2_ surfaces with oxygen vacancies by roughly 0.10 eV, in comparison to surfaces without these vacancies. The decrease in adsorption energy indicates a weaker interaction between the Rh_3_ clusters and the TiO_2_ surface in the presence of oxygen vacancies, which could be associated with alterations in the electronic structure of the substrate. This result is crucial because it emphasizes how the existence of oxygen vacancies might impact the stability and effectiveness of metal clusters that are adsorbed, potentially influencing the catalytic performance in applications such as photocatalysis and pollutant degradation. Further comprehension of these interactions could potentially result in the development of more customized catalyst designs that enhance performance by optimizing certain surface features. Future research might prioritize studying the precise processes via which oxygen vacancies impact surface contacts and experiment with various cluster arrangements to enhance catalytic efficiency.

**Figure 4:**
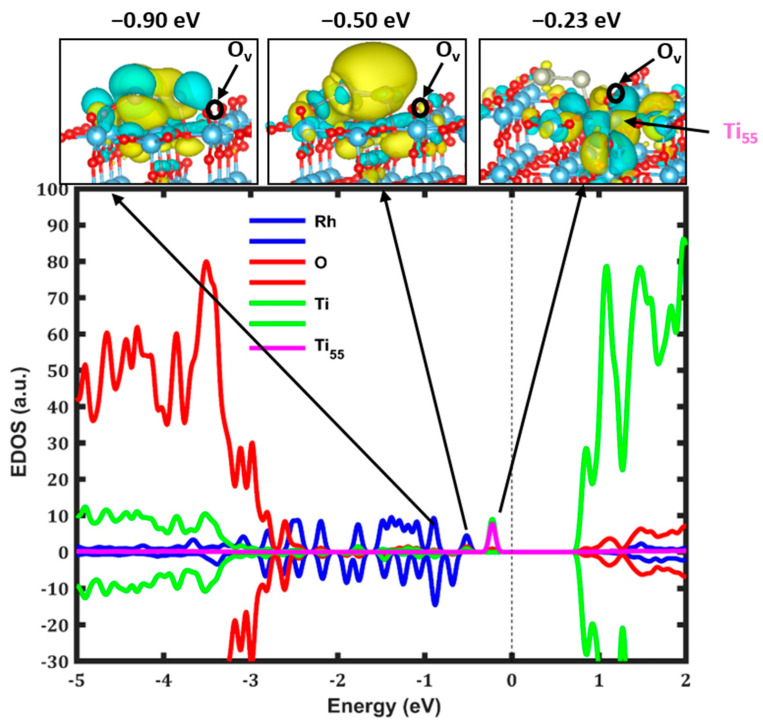
Projected density of states and wavefunctions for the most stable structure of the Rh_3_ cluster adsorbed on reduced TiO_2_. The states linked to Ti, Rh, O, and Ti_55_ orbitals are shown by green, blue, red, and pink colors, respectively. The black circles (O_v_) show the oxygen vacancy position.

The results shown in Figure 4 underscore the profound modifications in the electronic characteristics of TiO_2_, instigated by its interaction with the Rh_3_ cluster, alongside the introduction of the oxygen vacancy. A pivotal finding from this research is the formation of intragaps within the band gap, especially a polaronic state at −0.23 eV linked to the Ti_55_ atom located on the TiO_2_ surface, which arises due to the oxygen vacancy on the surface of TiO_2_. This polaronic state, along with the HOMO of the Rh_3_ cluster being identified at an elevated energy level of about −0.50 eV, marks a substantial shift in the electronic characteristics of TiO_2_. Additionally, the identification of an apparent state at −0.90 eV, associated with the orbitals of Ti and O, as well as the states localized on the Rh cluster, indicates a complex restructuring of electronic states. These dispersed states could significantly influence the photocatalytic performance of rutile TiO_2_ (110) under visible light [55], pointing to the critical role of both the oxygen vacancy and the Rh_3_ cluster in optimizing the energy efficiency of water splitting processes. Furthermore, the Rh_3_ donates approximately +0.19 e^−^ to the TiO_2_ surface. When contrasted with configurations without an oxygen vacancy (illustrated in Figure 2c), this electron donation is reduced by 0.37 e^−^. The formation of a polaron, a direct consequence of this electron donation, plays a crucial role in improving the absorption of photons in the visible light range, which is a critical component for effective photocatalysis. The simultaneous integration of the Rh_3_ cluster and an oxygen vacancy not only modulates the electronic structure but potentially lowers the threshold energy required for catalytic reactions, highlighting a promising avenue for enhancing photocatalytic performance in water splitting applications.

In light of the comprehensive examination of the interactions between the Rh_3_ clusters and rutile TiO_2_ (110) surfaces for the production of clean hydrogen, subsequent investigations should delve into the accumulative effect of additional clusters, such as Pt or Pd, to amplify photocatalytic performance. Furthermore, carrying out comparative analyses using single Rh atoms, Rh dimers, Rh_7_ and Rh_9_ clusters, and Rh nanoparticles on TiO_2_ would enhance our comprehension of the catalytic effects that vary with size. Moreover, exploring variations in the surface architecture and oxygen vacancy concentration of TiO_2_ is expected to yield profound insights into the optimization of photocatalytic efficiency. The confluence of experimental verification with computational studies will prove essential in propelling the real-world utility of these configurations in clean energy technologies. This might entail the empirical assessment of various cluster configurations and dimensions on TiO_2_ substrates in actual operational scenarios to evaluate their photocatalytic efficacy and longevity. Future research that includes band structure and DOS investigations will provide a more thorough understanding of the electronic changes brought about by Rh_3_ loading and how these affect photocatalytic activity. This method opens the door for important developments in material science and photocatalysis by contributing to a greater understanding of material properties and how to optimize them for improved photocatalytic efficiency. It also promises to confirm and expand on our existing discoveries. This exploration is poised to usher in breakthroughs in material science and photocatalysis, thereby facilitating the fabrication of more efficacious and durable photocatalysts for green hydrogen generation, making substantial contributions to renewable energy paradigms.

## 4. Concluding Remarks

This research marks a significant advance in the field of solar-driven hydrogen production, highlighting the innovative use of Rh_3_ clusters on rutile TiO_2_ to enhance photocatalytic activity. Through the application of sophisticated computational techniques, specifically DFT combined with HF, this study examines the structural and electronic characteristics of Rh_3_ clusters, both in the gas phase and when coupled with pristine and reduced TiO_2_ surfaces. The results indicate that Rh_3_ clusters boost the photocatalytic performance of TiO_2_ by modifying its electronic structures and expanding its light absorption capacity, especially in the visible light region. This improvement is obvious from the generation of intragaps, particularly localized states, alongside an enhanced mechanism for charge transfer, which is vital for the efficacy of photocatalytic processes. Additionally, this research evaluates the stability of various configurations of Rh_3_ clusters on TiO_2_ surfaces, highlighting the critical role of cluster orientation and the physical configuration of the surface in optimizing photocatalytic performance. The most stable adsorption configurations of Rh_3_ result in a charge donation of about −0.56 e^−^ to TiO_2_. Moreover, the integration of the Rh_3_ cluster onto the TiO_2_ (110) surface is demonstrated to boost the stability of the material, as demonstrated by a 0.10 eV increment in the oxygen vacancy formation energy. The localized state, lying 0.8 eV below the CB edge (as shown in Figure 4), has a high energy level. This may substantially improve the process of photocatalytic water splitting, leading to the efficient synthesis of green hydrogen. The location of this state improves the photocatalyst’s capacity to efficiently transfer electrons. During the process of water splitting, the electrons in the polaronic state can be energized and move to the CB, resulting in the creation of holes. Subsequently, these cavities can engage in the process of water oxidation, resulting in the generation of oxygen. The energized electrons in the CB have the ability to decrease the number of protons in water, resulting in the production of hydrogen. Hence, the precise location of the polaron level plays a vital role in ensuring effective photocatalytic performance, thereby impacting the HER in water splitting protocols.

This research underscores the critical role of Rh_3_ clusters in augmenting the photocatalytic capabilities of TiO_2_, with significant implications for renewable energy generation, notably in the context of hydrogen generation from H_2_O. It delves into the electronic behavior and stability of clusters on metal oxides, offering strategic directions for the creation of new materials aimed at sustainable energy solutions. Utilizing DFT computations alongside analyses of cluster-surface interactions, this study establishes a benchmark for subsequent studies. Future research endeavors might investigate a variety of metal–semiconductor combinations, explore different surface architectures, and integrate both experimental and theoretical methodologies to enhance the efficiency of photocatalytic systems for practical deployment.

## Figures and Tables

**Table 1:** Adsorption energies ($E_{ads}$) determined via DFT and the Bader charge analysis for triangular Rh_3_ clusters, as depicted in Figure 2.

Structure	Figure 2a	Figure 2b	Figure 2c
*E_ads_* (eV)	−2.45	−2.25	−4.36
Charge on Rh_3_ (e^−^)	+0.71	+0.76	+0.56

## Data Availability

Data is contained within the article and Supplementary Materials.

## Supplementary Materials

The following supporting information can be downloaded at: https://www.mdpi.com/article/10.3390/nano14121051/s1, Figure S1: Projected density of states of pristine rutile TiO_2_ (110); Figure S2: Oxygen vacancy formation at (a) surface and (b) subsurface locations of TiO_2_ rutile (110); Figure S3: Oxygen vacancy formation at (a) top view and (b) lateral view of the most stable Ru_3_@TiO_2_ rutile (110); Table S1: Comparisons of formation energies of oxygen vacancy for structures shown in Figures S2 and Figure S3. Reference [25] is cited in the supplementary materials.

## References

[1] Dunn S., Verne J., Mazza P. (2002). Futures: Weather: By Guy Dauncey with in order to make them. Int. J. Hydrogen Energy.

[2] Eberle U., Müller B., von Helmolt R. (2012). Fuel cell electric vehicles and hydrogen infrastructure: Status 2012. Energy Environ. Sci..

[3] Acar C., Dincer I. (2014). Comparative assessment of hydrogen production methods from renewable and non-renewable sources. Int. J. Hydrogen Energy.

[4] Thomas C.E. (2009). Fuel cell and battery electric vehicles compared. Int. J. Hydrogen Energy.

[5] Mazloomi K., Gomes C. (2012). Hydrogen as an energy carrier: Prospects and challenges. Renew. Sustain. Energy Rev..

[6] Maeda K. (2011). Photocatalytic water splitting using semiconductor particles: History and recent developments. J. Photochem. Photobiol. C Photochem. Rev..

[7] Fujishima A., Honda K. (1972). Electrochemical Photolysis of Water at a Semiconductor Electrode. Nature.

[8] AMurphy A., Barnes P., Randeniya L., Plumb I., Grey I., Horne M., Glasscock J. (2006). Efficiency of solar water splitting using semiconductor electrodes. Int. J. Hydrogen Energy.

[9] Fujishima A., Rao T.N., Tryk D.A. (2000). Titanium dioxide photocatalysis. J. Photochem. Photobiol. C.

[10] YTezuka Y., Shin S., Ishii T., Ejima T., Suzuki S., Sato S. (1994). Photoemission and Bremsstrahlung Isochromat Spectroscopy Studies of TiO_2_ (Rutile) and SrTiO_3_. J. Phys. Soc. Jpn..

[11] Klein M., Nadolna J., Gołąbiewska A., Mazierski P., Klimczuk T., Remita H., Zaleska-Medynska A. (2016). The effect of metal cluster deposition route on structure and photocatalytic activity of mono- and bimetallic nanoparticles supported on TiO_2_ by radiolytic method. Appl. Surf. Sci..

[12] Zhang H., Ming H., Lian S., Huang H., Li H., Zhang L., Liu Y., Kang Z., Lee S.-T. (2011). Fe_2_O_3_/carbon quantum dots complex photocatalysts and their enhanced photocatalytic activity under visible light. Dalton Trans..

[13] Chainarong S., Sikong L., Pavasupree S., Niyomwas S. (2011). Synthesis and characterization of nitrogen-doped TiO_2_ nanomaterials for photocatalytic activities under visible light. Energy Procedia.

[14] Fujii H., Inata K., Ohtaki M., Eguchi K., Arai H. (2001). Synthesis of TiO_2_/CdS nanocomposite via TiO_2_ coating on CdS nanoparticles by compartmentalized hydrolysis of Ti alkoxide. J. Mater. Sci..

[15] Mba M., D’acunzo M., Salice P., Carofiglio T., Maggini M., Caramori S., Campana A., Aliprandi A., Argazzi R., Bignozzi C.A. (2013). Sensitization of nanocrystalline TiO_2_ with multibranched organic dyes and co(III)/(II) Mediators: Strategies to improve charge collection efficiency. J. Phys. Chem. C.

[16] Kudo A., Miseki Y. (2009). Heterogeneous photocatalyst materials for water splitting. Chem. Soc. Rev..

[17] Perron H., Domain C., Roques J., Drot R., Simoni E., Catalette H. (2007). Optimisation of accurate rutile TiO_2_ (110), (100), (101) and (001) surface models from periodic DFT calculations. Theor. Chem. Acc..

[18] Ramamoorthy M., Vanderbilt D., King-Smith R.D. (1994). First-principles calculations of the energetics of stoichiometric TiO_2_ surfaces. Phys. Rev. B.

[19] Diebold U. (2002). The surface science of titanium dioxide. Surf. Sci. Rep..

[20] Yu J., Qi L., Jaroniec M. (2010). Hydrogen production by photocatalytic water splitting over Pt/TiO_2_ nanosheets with exposed (001) facets. J. Phys. Chem. C.

[21] Sclafani A., Herrmann J.-M. (1998). Influence of metallic silver and of platinum-silver bimetallic deposits on the photocatalytic activity of titania (anatase and rutile) in organic and aqueous media. J. Photochem. Photobiol. A Chem..

[22] Haruta M., Daté M. (2001). Advances in the catalysis of Au nanoparticles. Appl. Catal. A Gen..

[23] Alotaibi M. (2024). Geometrical Stabilities and Electronic Structures of Ru_3_ Clusters on Rutile TiO_2_ for Green Hydrogen Production. Nanomaterials.

[24] de Lara-Castells M.P., Cabrillo C., Micha D.A., Mitrushchenkov A.O., Vazhappilly T. (2018). Ab initio design of light absorption through silver atomic cluster decoration of TiO_2_. Phys. Chem. Chem. Phys..

[25] Alotaibi M., Wu Q., Lambert C. (2023). Computational studies of Ag_5_ atomic quantum clusters deposited on anatase and rutile TiO_2_ surfaces. Appl. Surf. Sci..

[26] Gao P., Yang L., Xiao S., Wang L., Guo W., Lu J. (2019). Effect of Ru, Rh, Mo, and Pd adsorption on the electronic and optical properties of anatase TiO_2_(101): A DFT investigation. Materials.

[27] Jin C., Dai Y., Wei W., Ma X., Li M., Huang B. (2017). Effects of single metal atom (Pt, Pd, Rh and Ru) adsorption on the photocatalytic properties of anatase TiO_2_. Appl. Surf. Sci..

[28] Wang J., Liu K., Zhang B., Qiu Y., Xiang Y., Lin W., Yang B., Li B., Ma G. (2021). Doping rh into TiO_2_ as a visible-light-responsive photocatalyst: The difference between rutile and anatase. Appl. Phys. Lett..

[29] Albuquerque B.L., Chacón G., Nazarkovsky M., Dupont J. (2020). Rhodium nanoparticles impregnated on TiO_2_: Strong morphological effects on hydrogen production. New J. Chem..

[30] Seriani N., Pinilla C., Crespo Y. (2015). Presence of gap states at Cu/TiO_2_ anatase surfaces: Consequences for the photocatalytic activity. J. Phys. Chem. C.

[31] López-Caballero P., Hauser A.W., de Lara-Castells M.P. (2019). Exploring the Catalytic Properties of Unsupported and TiO_2_-Supported Cu_5_ Clusters: CO_2_ Decomposition to CO and CO_2_ Photoactivation. J. Phys. Chem. C.

[32] de Lara-Castells M.P., Hauser A.W., Ramallo-López J.M., Buceta D., Giovanetti L.J., López-Quintela M.A., Requejo F.G. (2019). Increasing the optical response of TiO_2_ and extending it into the visible region through surface activation with highly stable Cu_5_ clusters. J. Mater. Chem. A.

[33] Lin Y., Jiang Z., Zhu C., Hu X., Zhu H., Zhang X., Fan J., Lin S.H. (2013). The optical absorption and hydrogen production by water splitting of (Si,Fe)-codoped anatase TiO_2_ photocatalyst. Int. J. Hydrogen Energy.

[34] Majumdar D., Balasubramanian K. (1997). Theoretical studies of CO interaction on Rh_3_ cluster. J. Chem. Phys..

[35] Huang J., Li G., Zhou Z., Jiang Y., Hu Q., Xue C., Guo W. (2018). Efficient photocatalytic hydrogen production over Rh and Nb codoped TiO_2_ nanorods. Chem. Eng. J..

[36] Camposeco R., Hinojosa-Reyes M., Zanella R. (2021). Highly efficient photocatalytic hydrogen evolution by using Rh as co-catalyst in the Cu/TiO_2_ system. Int. J. Hydrogen Energy.

[37] Subramanian V., Wolf E.E., Kamat P.V. (2004). Catalysis with TiO_2_/Gold Nanocomposites. Effect of Metal Particle Size on the Fermi Level Equilibration. J. Am. Chem. Soc..

[38] Assadi M.H.N., Hanaor D.A. (2016). The effects of copper doping on photocatalytic activity at (101) planes of anatase TiO_2_: A theoretical study. Appl. Surf. Sci..

[39] Iwashina K., Kudo A. (2011). Rh-doped SrTiO_3_ photocatalyst electrode showing cathodic photocurrent for water splitting under visible-light irradiation. J. Am. Chem. Soc..

[40] Nørskov J.K., Bligaard T., Logadottir A., Kitchin J.R., Chen J.G., Pandelov S., Stimming U. (2005). Trends in the Exchange Current for Hydrogen Evolution. J. Electrochem. Soc..

[41] Alotaibi M. (2024). Geometrical Stabilities and Electronic Structures of Rh_5_ Nanoclusters on Rutile TiO_2_ (110) for Green Hydrogen Production. Nanomaterials.

[42] Heyd J., Scuseria G.E. (2006). Influence of the exchange screening parameter on the performance of screened hybrid functionals. J. Chem. Phys..

[43] Kresse G., Furthmüller J. (1996). Efficiency of ab-initio total energy calculations for metals and semiconductors using a plane-wave basis set. Comput. Mater. Sci..

[44] Kresse G., Furthmüller J. (1996). Efficient iterative schemes for ab initio total-energy calculations using a plane-wave basis set. Phys. Rev. B.

[45] Kresse G., Hafner J. (1994). Ab initio molecular-dynamics simulation of the liquid-metalamorphous- semiconductor transition in germanium. Phys. Rev. B.

[46] Perdew J.P., Burke K., Ernzerhof M. (1996). Generalized gradient approximation made simple. Phys. Rev. Lett..

[47] Kresse G., Joubert D. (1999). From ultrasoft pseudopotentials to the projector augmented-wave method. Phys. Rev. B.

[48] Blöchl P.E. (1994). Projector augmented-wave method. Phys. Rev. B.

[49] Morgan B.J., Watson G.W. (2007). A DFT + U description of oxygen vacancies at the TiO_2_ rutile (1 1 0) surface. Surf. Sci..

[50] Morgan B.J., Watson G.W. (2009). A density functional theory + u study of Oxygen vacancy formation at the (110), (100), (101), and (001) surfaces of rutile TiO_2_. J. Phys. Chem. C.

[51] Antony A., Hakanoglu C., Asthagiri A., Weaver J.F. (2012). Dispersion-corrected density functional theory calculations of the molecular binding of n-alkanes on Pd(111) and PdO(101). J. Chem. Phys..

[52] Grimme S., Antony J., Ehrlich S., Krieg H. (2010). A consistent and accurate ab initio parametrization of density functional dispersion correction (DFT-D) for the 94 elements H-Pu. J. Chem. Phys..

[53] Momma K., Izumi F. (2011). VESTA 3 for three-dimensional visualization of crystal, volumetric and morphology data. J. Appl. Crystallogr..

[54] Ren Y., Han Q., Su Q., Yang J., Zhao Y., Wen H., Jiang Z. (2021). Effects of 4d transition metals doping on the photocatalytic activities of anatase TiO_2_ (101) surface. Int. J. Quantum Chem..

[55] López-Caballero P., Miret-Artés S., Mitrushchenkov A.O., de Lara-Castells M.P. (2020). Ag_5_-induced stabilization of multiple surface polarons on perfect and reduced TiO_2_ rutile (110). J. Chem. Phys..

[56] Gomes Silva C., Juárez R., Marino T., Molinari R., García H. (2011). Influence of Excitation Wavelength (UV or Visible Light) on the Photocatalytic Activity of Titania Containing Gold Nanoparticles for the Generation of Hydrogen or Oxygen from Water. J. Am. Chem. Soc..

[57] Pabisiak T., Kiejna A. (2007). Energetics of oxygen vacancies at rutile TiO_2_(110) surface. Solid State Commun..

[58] Oviedo J., Miguel M.A.S., Sanz J.F. (2004). Oxygen vacancies on TiO_2_(110) from first principles calculations. J. Chem. Phys..

